# Genome-Wide Sequence and Expression Analysis of the NAC Transcription Factor Family in Polyploid Wheat

**DOI:** 10.1534/g3.117.043679

**Published:** 2017-07-11

**Authors:** Philippa Borrill, Sophie A. Harrington, Cristobal Uauy

**Affiliations:** Department of Crop Genetics, John Innes Centre, Norwich NR4 7UH, UK

**Keywords:** wheat, transcription factors, NAC, phylogenetics, gene expression

## Abstract

Many important genes in agriculture correspond to transcription factors (TFs) that regulate a wide range of pathways from flowering to responses to disease and abiotic stresses. In this study, we identified 5776 TFs in hexaploid wheat (*Triticum aestivum*) and classified them into gene families. We further investigated the NAC family exploring the phylogeny, C-terminal domain (CTD) conservation, and expression profiles across 308 RNA-seq samples. Phylogenetic trees of NAC domains indicated that wheat NACs divided into eight groups similar to rice (*Oryza sativa*) and barley (*Hordeum vulgare*). CTD motifs were frequently conserved between wheat, rice, and barley within phylogenetic groups; however, this conservation was not maintained across phylogenetic groups. Three homeologous copies were present for 58% of NACs, whereas evidence of single homeolog gene loss was found for 33% of NACs. We explored gene expression patterns across a wide range of developmental stages, tissues, and abiotic stresses. We found that more phylogenetically related NACs shared more similar expression patterns compared to more distant NACs. However, within each phylogenetic group there were clades with diverse expression profiles. We carried out a coexpression analysis on all wheat genes and identified 37 modules of coexpressed genes of which 23 contained NACs. Using gene ontology (GO) term enrichment, we obtained putative functions for NACs within coexpressed modules including responses to heat and abiotic stress and responses to water: these NACs may represent targets for breeding or biotechnological applications. This study provides a framework and data for hypothesis generation for future studies on NAC TFs in wheat.

Transcription factors (TFs), by virtue of their role in activating or repressing gene expression, regulate many biological processes. They are particularly important to agriculture because TFs have been identified to be the causal genes underlying agronomic traits including flowering time, nutrient content, and stress responses (Yan *et al.* 2003; Uauy *et al.* 2006; Jensen and Skriver 2014). As such, identifying and characterizing the TFs in crops provides an important first step to engineer strategies for the improvement of agriculturally important traits.

Wheat is the most widely grown crop globally, providing roughly 20% of the daily calorific intake and 25% of protein intake worldwide (www.fao.org/faostat). The economic importance of wheat is also great, comprising over 40% of global cereal trade (FAO 2017). Twin pressures of increasing global population and changing climatic conditions make it ever more urgent that novel wheat varieties are developed that have improved yield potential, end-use quality, and increased tolerances to biotic and abiotic stresses, such as drought and heat.

Of the many TF families, the plant-specific NAC family has been shown to regulate several biological processes in wheat. Named after the first three such TFs identified [NAM, ATAF1/2 (Souer *et al.* 1996), and CUC2 (Aida *et al.* 1997)], the NAC TF family is characterized by a highly conserved NAC domain, typically at the N-terminal region, often followed by an intrinsically disordered transcriptional regulatory domain at the C-terminal region that is poorly conserved (Ernst *et al.* 2004; Olsen *et al.* 2005; Xie *et al.* 2000). The NAC domain is well characterized, and is required for protein–DNA interactions (Welner *et al.* 2012; Xie *et al.* 2000) and protein dimerization (Ernst *et al.* 2004). In wheat, NAC TFs are known to be involved in processes such as senescence and nutrient remobilization (Uauy *et al.* 2006; Zhao *et al.* 2015) as well as responses to abiotic and biotic stresses, ranging from stripe rust (Feng *et al.* 2014; Xia *et al.* 2010a,b; Wang *et al.* 2015) to abiotic stresses including drought (Huang *et al.* 2015; Tang *et al.* 2012; Xue *et al.* 2006; Mao *et al.* 2014, 2012) and salt tolerance (Huang *et al.* 2015; Mao *et al.* 2014, 2012). The phylogenetic relationships of NAC TFs in different species have been identified and used to characterize evolutionarily-conserved groupings of NAC TFs (Ooka *et al.* 2003; Pereira-Santana *et al.* 2015; Shen *et al.* 2009). However, until recently, such an analysis was hindered in wheat due to the lack of a high-quality reference genome sequence and a comprehensive set of gene models.

Recent advances in wheat genomics now provide the opportunity to characterize TF families much more completely in wheat (Uauy 2017). In this study, we used the recently published high-quality TGAC gene models (Clavijo *et al.* 2017) to annotate all characterized TF families in wheat, and compare their abundance with other previously characterized crop species and wild relatives of wheat. We focused on the NAC TF family to understand the evolutionary relationships within the family itself and global expression patterns using large-scale RNA-seq studies (Borrill *et al.* 2016; Clavijo *et al.* 2017) and coexpression networks. The analyses presented in this study allow novel hypotheses to be generated to predict TF function and pave the way for future functional characterization.

## Materials and Methods

### Annotation of TFs

We downloaded the protein sequences for the gene models produced for the TGAC wheat assembly (Clavijo *et al.* 2017) from EnsemblPlants release-32 (Bolser *et al.* 2015) (http://plants.ensembl.org/index.html). These contained 249,547 transcripts corresponding to 195,864 genes of which 104,091 were high and 91,773 low confidence. We used these sequences to identify putative TFs using three methods for both high- and low-confidence genes. The use of these gene models as the starting point for TF annotation means that any TFs without a gene model in the TGAC wheat assembly were not considered in this analysis.

#### BLAST-based approach:

We downloaded the protein sequences of TFs annotated in PlantTFDBv3.0 (Jin *et al.* 2014) for *Aegilops tauschii*, *Hordeum vulgare*, *Oryza sativa* subsp. *japonica*, *O. sativa* subsp. *indica*, *Triticum urartu*, and *T. aestivum* (from ESTs Unigene Build #63). We performed a blastp analysis of these protein sequences against the TGAC wheat protein sequences downloaded from EnsemblPlants with the parameter -max_target_sequations 10 to retrieve the top 10 hits. We combined the BLAST results from each of the six species and removed duplicate genes.

#### Ensembl orthologs-based approach:

We used EnsemblPlants Biomart to download wheat orthologs to the TFs in five species (*A. tauschii*, *H. vulgare*, *O. sativa* subsp. *japonica*, *O. sativa* subsp. *indica*, and *T. urartu*), which were available on EnsemblPlants and annotated in PlantTFDBv3.0. For *O. sativa* subsp. *japonica* (before downloading wheat orthologs) we converted the MSU nomenclature rice gene identifiers from PlantTFDB to RAP rice gene identifiers, which were compatible with EnsemblPlants using the RAPD converter http://rapdb.dna.affrc.go.jp/tools/converter/run. This step retained 1816 RAP genes out of 1859 MSU genes originally identified by PlantTFDB.

#### TGAC functional annotation approach:

We searched the functional annotation available for the TGAC wheat assembly (Clavijo *et al.* 2017) for all genes with PFAMs associated with TFs. The PFAMs associated with TFs were obtained from PlantTFDB.

### Generating a combined list of TFs

To generate a reliable list of TFs for wheat, we combined the lists of genes identified by the blastp, Ensembl ortholog, and functional annotation approaches. This included 9416 genes (13,325 transcripts). This list may include genes that are not TFs in wheat due to changes to their sequences from their orthologs in the other monocot species or because genes with certain combinations of PFAM domains are known not to act as TFs (Jin *et al.* 2014). Therefore, we ran the 13,325 transcripts identified through the PlantTFDBv3.0 prediction server http://planttfdb_v3.cbi.pku.edu.cn/prediction.php in batches of 1000 genes. This resulted in the annotation of, in total, 7415 genes (10,303 transcripts), of which 5776 genes (8609 transcripts) were from high-confidence gene models. PlantTFDBv3.0 also assigned TFs to TF families.

### NAC TF homeologs and orthologs

From the list of TFs identified, we extracted genes that were classified as NACs by PlantTFDBv3.0. For further analysis, we selected only NACs with high-confidence gene models (453/574). For these 453 high-confidence NAC genes, we downloaded information about wheat homeologs from EnsemblPlants Biomart and grouped them into triads (A, B, and D genome homeologs). Homeologs were calculated by EnsemblPlants using a pipeline based on Vilella *et al.* (2009) with updated information available from http://plants.ensembl.org/info/genome/compara/homology_method.html. Rice (*O. sativa* subsp. *japonica*) and barley orthologs were identified by reciprocal BLAST of coding sequences. If the reciprocal BLAST did not identify the same pair of genes in both directions, they were not considered orthologs.

### Phylogenetic tree generation and NAC group assignment

We aligned the NAC protein sequences with Clustal Omega v1.2.0 (Sievers *et al.* 2011) using default settings. We kept only the NAC domain from the start of subdomain A to the end of subdomain E (Ooka *et al.* 2003; Uauy *et al.* 2006) to create phylogenetic trees for wheat, barley, and rice NACs. After manual inspection, we found that a few regions within the NAC domain alignment were poorly conserved with amino acids only present in a few sequences. For this reason, we only retained amino acid positions that were present in ≥ 10% of sequences. We removed any sequences that did not contain any NAC domain sequence. We used RAxML v8.2.1 (Stamatakis 2014) to create maximum likelihood phylogenetic trees using the auto setting to detect the best protein model, 100 maximum likelihood searchers, and 100 rapid bootstraps.

The barley and rice NACs had already been assigned to groups a–h in Christiansen *et al.* (2011) and Shen *et al.* (2009), respectively. Wheat genes that were phylogenetically grouped with barley or rice genes with a group classification were assigned to the appropriate group. In cases where the specific barley or rice ortholog belonged to a group dissimilar to the rest of the clade, the wheat genes were not assigned to a group (23 genes). In total, 430 wheat genes were assigned to a group. Figures with the groups alongside the NAC phylogeny were created using iTOL (Letunic and Bork 2016). We reran RAxML to make an individual phylogeny for groups a–h for wheat NACs and, separately, wheat, barley, and rice NACs.

### CTD motif discovery

We carried out *de novo* analysis of motifs in the a–h NAC TF groups using the MEME program (version 4.9.1) (Bailey *et al.* 2009). For each group, a maximum of 10 motifs were identified that occurred in all sequences and were between 5 and 20 residues long. From these motifs, we considered the most significant motif for further analysis, as well as additional significant motifs that shared sequence similarities with previously defined motifs (Ooka *et al.* 2003; Pereira-Santana *et al.* 2015; Shen *et al.* 2009).

To complement the *de novo* analysis, we screened all the wheat, barley, and rice NACs for motifs that were previously characterized (Ooka *et al.* 2003). A background amino acid frequency for wheat was obtained from the full set of peptide sequences from the TGAC gene models. We converted the motifs i–xiii from Ooka *et al.* (2003) into Regex expressions, and then converted into MEME motif format using IUPAC2MEME (v 4.9.1) from the MEME suite (Supplemental Material, Table S1). Using these motifs and the wheat amino acid background frequencies, we searched all genes in the set with FIMO (v 4.9.1) from the MEME suite. In some cases, the Ooka groupings contained more than one motif (groups ii, iv, and ix; Table S1). Genes were considered part of group ii or group iv if at least one of the motifs was present. However, as the motifs from group ix were already split to form groups x and xi, only genes that contained both ix motifs were assigned to the ix group. Plots of the CTD motifs alongside the NAC phylogeny were created using iTOL (Letunic and Bork 2016).

### Gene expression analysis

We downloaded count and transcript per million (tpm) gene expression values for previously mapped RNA-seq samples from www.wheat-expression.com (Borrill *et al.* 2016; Clavijo *et al.* 2017). We excluded samples from cytogenetic stocks (*e.g.*, nullitetrasomic lines) and from synthetic hexaploid wheat. This resulted in 308 RNA-seq samples from 15 individual studies being included in our analysis. We collated per transcript expression levels into per gene expression levels using the R package tximport v1.0.3 (Soneson *et al.* 2015). We filtered the data to only keep genes whose expression was over 0.5 tpm in at least three samples to eliminate very low-expressed genes. We also filtered the data to exclude low-confidence genes. We generated plots of phylogenetic trees with heatmaps of gene expression using the R package ggtree v1.4.20 (Yu *et al.* 2017).

### Coexpression analysis

We carried out coexpression analysis using the R package Weighted Gene Correlation Network Analysis (WGCNA) v1.51 (Langfelder and Horvath 2008). We used the function pickSoftThreshold to calculate that a soft-threshold power of six was appropriate for a signed hybrid network for our 308 samples. Due to the large number of genes in our analysis (91,403), we used the blockwiseModules method to calculate the coexpression network in two blocks using the parameters maxPOutliers = 0.05, mergeCutHeight = 0.15, deepSplit = 2, minModuleSize = 30, networkType = “signed hybrid,” maxBlockSize = 46,000, corType = “bicor,” corOptions = “use = “p,” and maxPOutliers = 0.05.”

### GO enrichment analysis

We used the R package GOseq v1.26.0 (Young *et al.* 2010) to determine whether GO terms were enriched within each coexpression module. We used Revigo (Supek *et al.* 2011) to summarize GO term enrichment for GO terms overrepresented with a Benjamini–Hochberg adjusted *P*-value < 0.05.

### Data availability

The supplemental materials contain the following data: Table S1, NAC protein CTD motifs identified by Ooka *et al.* (2003); Table S2, wheat TF family genes with gene model confidence levels; Table S3, wheat TF distribution across chromosomes; Table S4, wheat, barley, and rice NAC orthologs; Table S5, CTD motifs per gene for wheat, barley, and rice; Table S6, *de novo* motif discovery in NAC groups; Table S7, gene and TF module allocation by WGCNA coexpression analysis; Table S8, most overrepresented biological process GO terms in coexpression modules; Figure S1, maximum likelihood phylogeny of wheat, barley, and rice NAC TF proteins constructed using the NAC domain; Figure S2, extended version of Figure 3, showing conserved CTDs in wheat, rice, and barley NAC TFs; Figure S3, extended version of Figure 4, showing gene expression of wheat NAC TFs in the context of the phylogeny.

Interactive trees for Figure 2, Figure 3, Figure S1, and Figure S2 are available at http://itol.embl.de/shared/sophie_harrington

## Results

### Wheat TFs identified in the TGAC assembly

In total, we annotated 5776 high-confidence genes as TFs in wheat, which is a threefold increase compared to the previous wheat TF annotation available from PlantTFDB (Table 1). We identified on average 5.1 times more TFs than in other diploid Triticeae species. However, only 3.1 times more TFs were identified for rice, as would be expected for a comparison between a diploid and hexaploid species. The incomplete nature of the Triticeae species’ genomes compared to the highly contiguous genome assemblies of rice may explain the higher than expected ratio to monocots other than rice. The annotation of low-confidence genes was also carried out and a complete set of TFs in wheat is available in Table S2.

**Table 1:** Comparison of TFs identified in monocot species

		Transcription Factor		
Species	Ploidy	Transcripts*^a^*	Genes	Families
*Oryza sativa subsp. indica*	2×	1891	1891	56
*Oryza sativa subsp. japonica* (MSU)	2×	2408	1859	56
*Hordeum vulgare*	2×	2621	1198	56
*Aegilops tauschii*	2×	1439	1439	55
*Triticum urartu*	2×	888	888	50
*Triticum aestivum* (ESTs Unigene #63)	6×	1940	1940	56
*Triticum aestivum* (TGAC assembly high-confidence genes)	6×	8609	5776	56

EST, expressed sequence tag.

a Values are from PlantTFDB for all species except the *T. aestivum* TGAC assembly, which is from this study.

**Figure 1:**
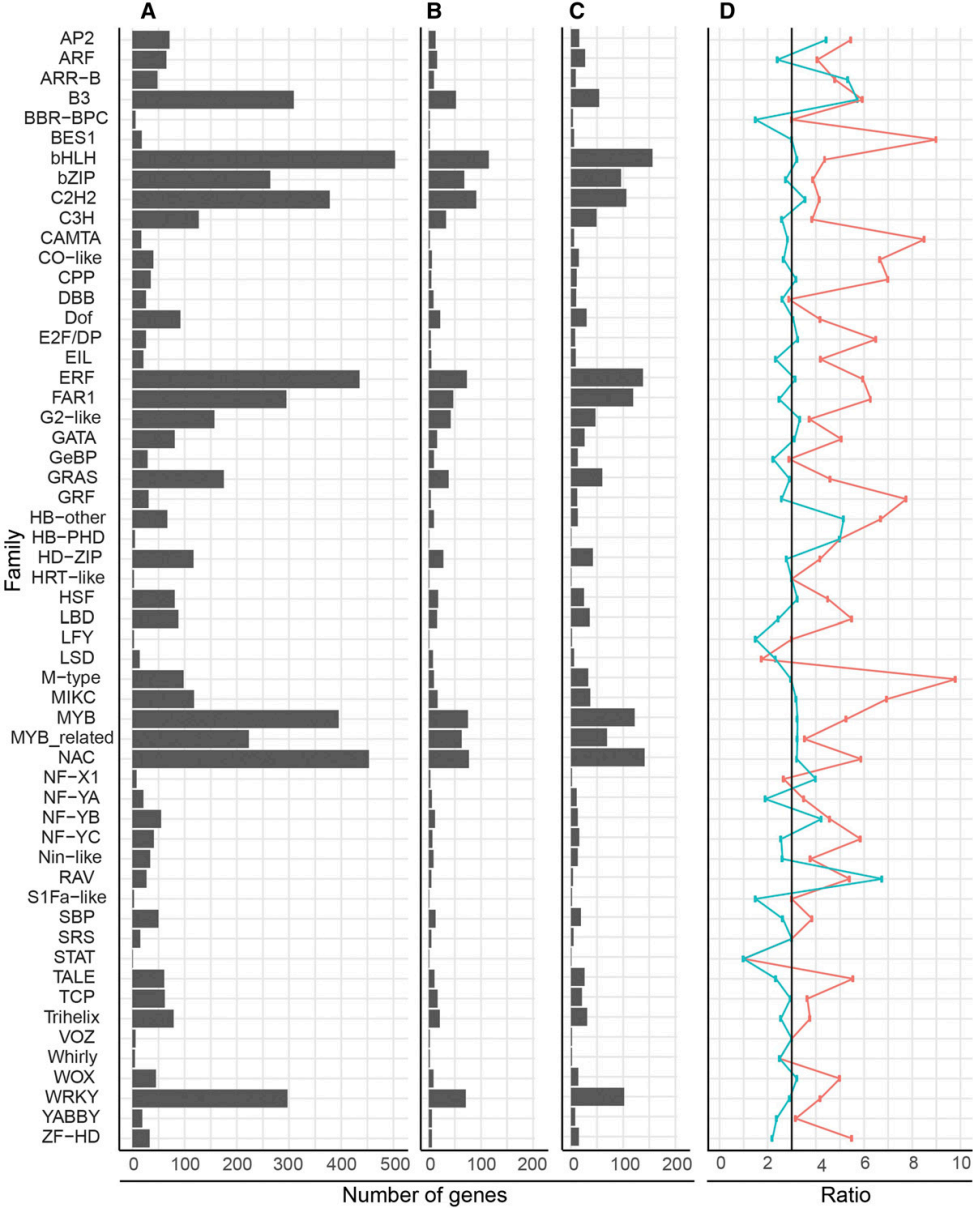
Comparison of genes identified per transcription factor family in wheat, barley, and rice. The number of genes in each family for (A) wheat, (B) barley, and (C) rice. (D) The ratio of wheat to barley (red) and wheat to rice (blue). In (D), the expected ratio (3:1) is indicated by a black line. Barley and rice data were obtained from PlantTFDBv3.0.

We found that distribution of TF families was similar between wheat, barley, and rice (Figure 1), with the largest families in all three species being bHLH and the smallest being STAT. In general, wheat had approximately three times more genes in each family than rice (Figure 1D, blue line). The only exceptions were the B3 and HB-other gene families, which were enriched in wheat with five times as many genes as in rice (χ^2^ test *P* < 0.001 and *P* = 0.048, respectively). The FAR1 family was the only family underrepresented in wheat with only 2.5 times as many genes as in rice (χ^2^ test *P* = 0.037). Compared to barley, most TF families had more members in wheat (Figure 1D, red line), which may be due to the incomplete nature of the barley genome.

We found that TFs were not distributed equally across all chromosomes, with group 1 and group 6 having an average of 223 and 206 TFs per homeolog, whereas group 3 and 5 had 300 and 304 TFs per homeolog, respectively (Table S3). Individual TF families differed from the global averages; for example NAC TFs were most frequent on chromosome groups 2 and 7 and least frequent on groups 1 and 6, whereas WRKY TFs were most frequent on groups 1 and 3 and least frequent on groups 4 and 6. We also found that, in general, slightly different numbers of each TF family were found on each homeolog.

### The NAC TF family in wheat, barley, and rice

We decided to focus our analysis on the NAC family of TFs, which is known to be involved in a range of agronomically relevant processes including abiotic and biotic stress responses. In total, we identified 453 NACs with high-confidence gene models using the PlantTFDBv3.0 classifications. We grouped the NACs into homeologous groups and identified their barley and rice orthologs by reciprocal blast (Table S4). Due to the hexaploid nature of wheat, genes are expected to be found as homeologous triads. We found that, of the 146 homeologous triads of NAC TFs, 58% had a single copy of each homeolog, while 33% of triads had a single copy of two homeologs with one homeolog absent. The remaining 9% of triads had variable numbers of homeologs retained. Therefore, in most cases, a single copy of each NAC TF has been retained, although one homeologous copy has been lost in one-third of triads.

**Figure 2:**
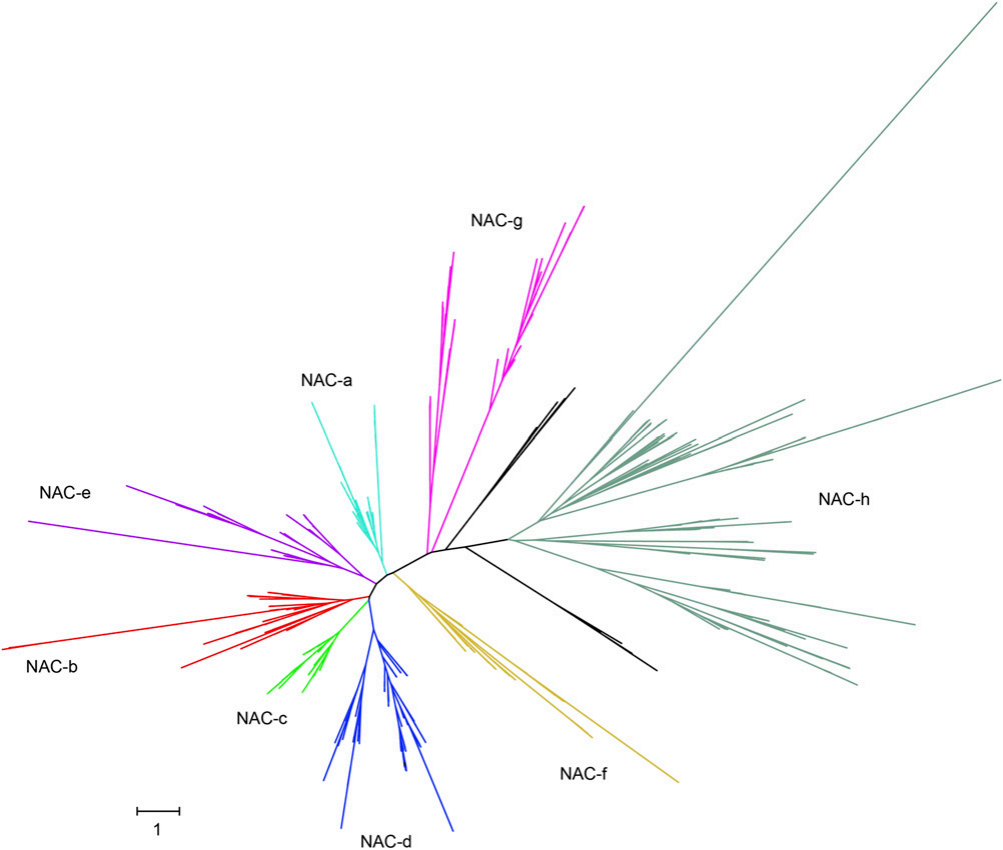
Maximum likelihood phylogeny of 667 NAC proteins from wheat, rice, and barley. The phylogeny was constructed using only the NAC domain. NAC groups a–h were assigned according to rice and barley orthologs. In cases where the group assigned to a rice or barley gene conflicted with the overall tree topology, no group was assigned (black branches). Details of individual genes are presented in Figure S1 and Table S4.

To understand more about NAC evolution in wheat, we generated a phylogenetic tree for wheat, barley, and rice NACs using their NAC domains (Figure 2). We used the closest related barley and rice NACs to assign wheat NACs into eight main groups (a–h) as proposed by Shen *et al.* (2009) (see Figure S1). In total, 430 NACs were assigned to groups while 23 NACs could not be assigned to a group (either the NAC group was different for a particular protein compared to the rest of the clade or there was no clear rice or barley ortholog). As expected, each group had in general three times more genes in wheat than in rice and barley. However, wheat has a reduced group f with only 13 genes compared to the 10 genes found in rice (χ^2^ *P* = 0.001), but not compared to barley. Groups e, g, and h are significantly enlarged in wheat compared to barley (χ^2^ *P* = 0.04, *P* = 0.04, and *P* < 0.001, respectively); however, the numbers of genes in each of these groups is lower in barley than in rice, which suggests that this trend is due to the incomplete barley genome rather than a true enrichment in wheat.

**Figure 3:**
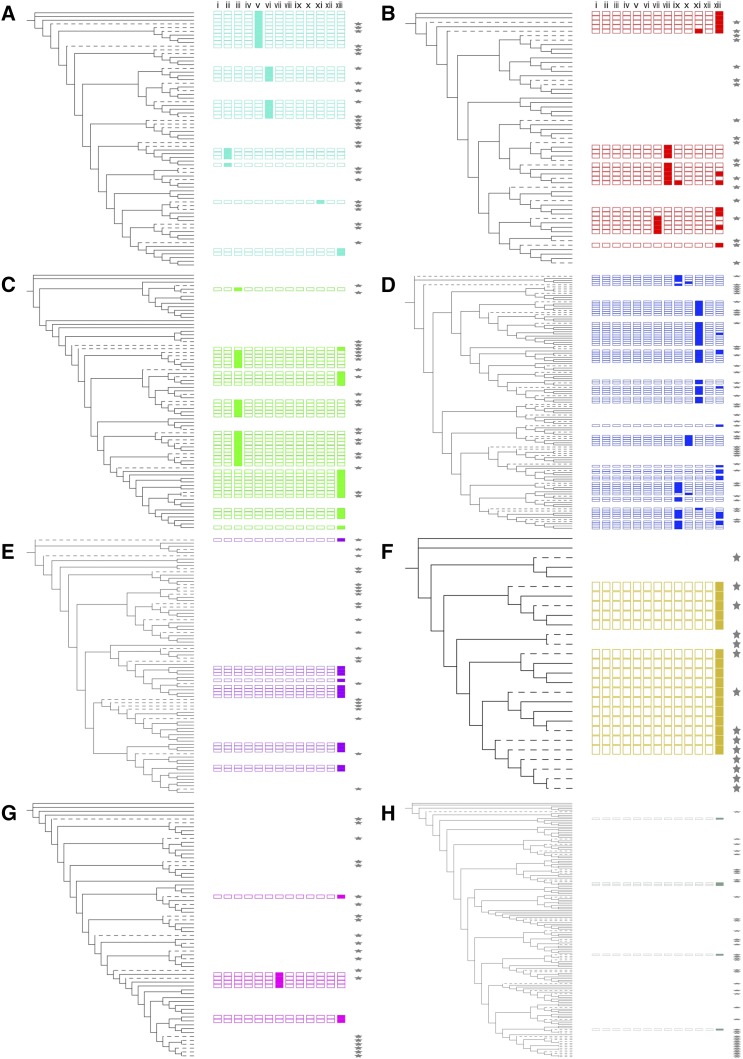
Conserved domains in the CTD of NAC transcription factors arranged by phylogenetic position. Known CTD motifs are shown alongside the wheat, barley, and rice NAC TFs for each group a–h (A–H), colored in accordance with Figure 2. Branches corresponding to wheat NAC TFs are solid black; those for rice and barley NAC TFs are dashed. Motifs are shown as boxes, matching (left to right) motifs i–xiii from Ooka *et al.* (2003). Motifs that are present in each protein (*P*-value < 0.05, *q*-value < 0.05) are shown by a solid-colored box, while absent motifs are shown by an empty outlined box. Genes with no significant motifs are shown with empty space. Barley and rice genes are indicated by the presence of a star to the right of the CTD motifs. Details are presented in Table S5, and the full phylogenetic tree is presented in Figure S2. CTD, C-terminal domain; TF, transcription factor.

We also investigated the less well characterized CTD, which is proposed to be a transcriptional activator or repressor (Tran *et al.* 2004; Yamaguchi *et al.* 2010; Kim *et al.* 2007). We found that previously identified CTD motifs (Ooka *et al.* 2003) were generally conserved between homeologs and were often conserved in specific clades within phylogenetic groups of wheat, barley, and rice NACs (Figure 3, Figure S2, and Table S5, http://itol.embl.de/shared/sophie_harrington). We found that 10 out of the 13 motifs previously identified were present in wheat, rice. and barley NACs. In general, each motif was predominantly found in one or two groups (*e.g.*, motifs ii, v, and vi were only in group a; motif vii in groups b and g; and motif viii in group b). However, motif xiii was found in proteins belonging to all groups. The presence of motifs was not equally distributed between the groups, with relatively few motifs in e, g, and h, and high frequency of motifs in c, d, and f. *De novo* motif discovery identified significant motifs shared by all genes within each group (Table S6). Of these motifs, six had been previously identified as NAC CTD motifs (Ooka *et al.* 2003; Pereira-Santana *et al.* 2015; Shen *et al.* 2009), while three represent novel motifs.

### NAC expression patterns relate to phylogenetic position

To explore the expression patterns of NAC TFs, we used publicly available gene expression data for 15 studies comprising 308 individual RNA-seq samples (Borrill *et al.* 2016; Clavijo *et al.* 2017). These samples included diverse developmental stages, tissues, and stress conditions including both biotic and abiotic stresses. We filtered the NAC genes to retain only genes expressed at over 0.5 tpm in at least three samples. Within the phylogenetic groups a–h there were 430 NACs, of which 356 passed this threshold. In most groups, the vast majority of NAC genes were expressed; however, in group h, only 50% of NACs were expressed in the conditions represented by the 308 RNA-seq samples.

**Figure 4:**
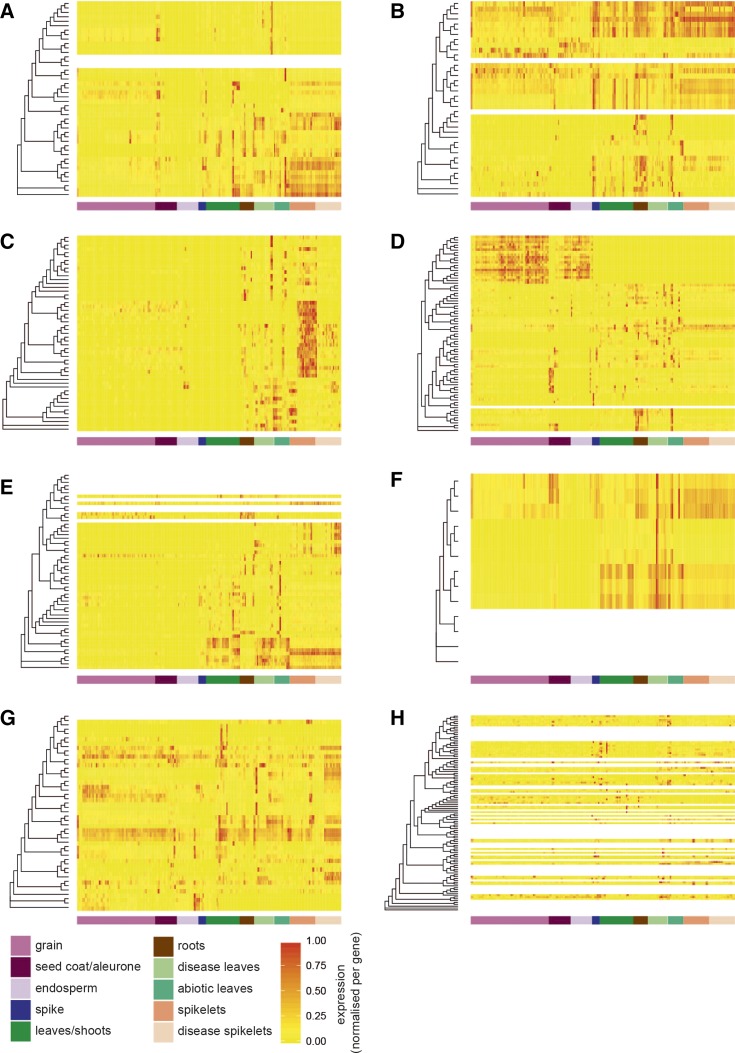
Relationship between phylogenetic position and NAC gene expression across 308 RNA-seq samples from diverse tissues, developmental stages, and stress conditions. The origin of each sample is indicated by the colored bar under each heatmap. Each panel (A–H) represents NAC genes belonging to that group according to the classification in Figure 2. Dendrograms indicate the maximum likelihood phylogeny of genes within each group. Genes that did not meet the minimum expression criteria (> 0.5 tpm in at least three samples) do not have expression data represented (white rows). All remaining expression data (tpm) was normalized per gene to range from 0 to 1. An extended version of the figure with the full phylogenetic trees is available as Figure S3. RNA-seq, RNA sequencing; tpm, transcripts per million.

We found that, in general, homeologs shared similar expression patterns across samples (Figure 4 and Figure S3). Gene expression patterns were more similar for genes found within the same phylogenetic group compared to genes in other groups. However, within each phylogenetic group, gene expression patterns were more highly conserved within closely related clades than across the whole group. These conserved expression clades often showed expression specific to particular tissues or environmental conditions. For example, in group d 18 genes form a subclade that is predominantly expressed in the grain and the endosperm (Figure 4D, uppermost genes), and in group c 20 genes form a clade that shows strong expression in spikelets, which is not seen in other group c genes (Figure 4C, middle). We did not observe a correlation between expression patterns and the presence of specific CTDs (data not shown).

**Figure 5:**
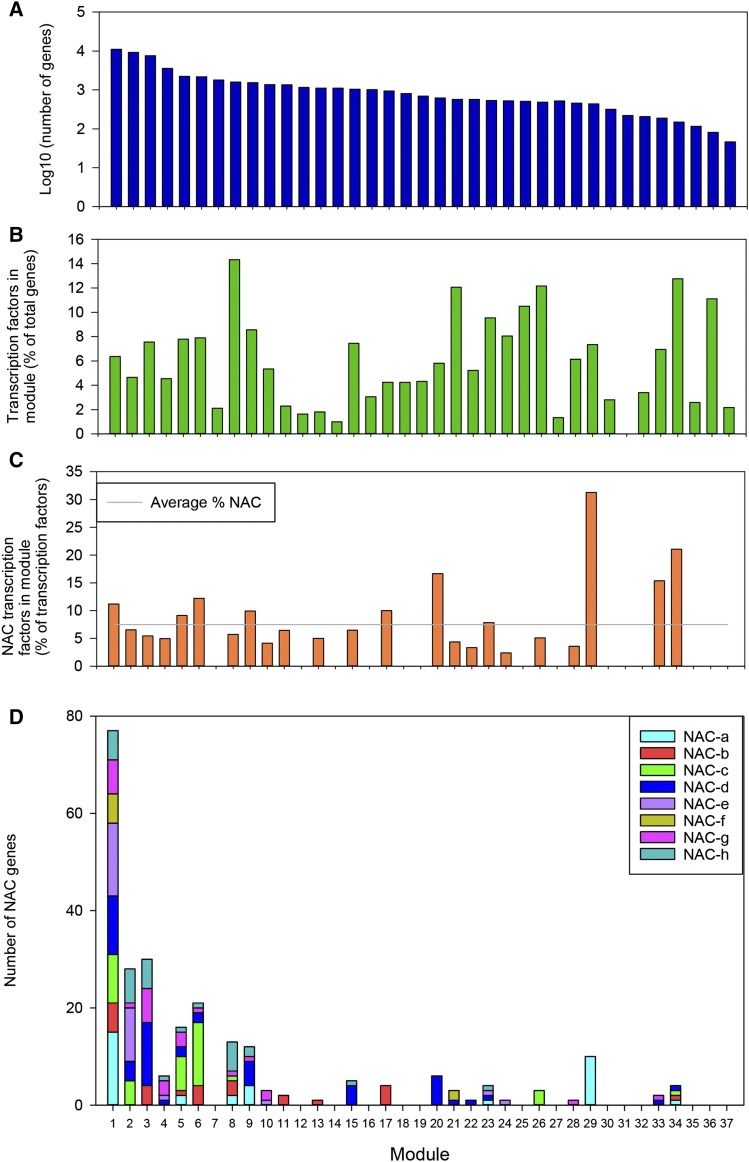
Distribution of genes and transcription factors (TFs) across modules. (A) Number of genes (log10), (B) percentage of genes that are TFs, (C) percentage of TFs that are NAC TFs, and (D) number of NACs from each phylogenetic group.

To explore the patterns of NAC TF expression in a global context, we carried out coexpression analysis using WGCNA across all gene families using the 308 RNA-seq samples. We could assign 61,325 genes (out of 91,403) to 37 coexpression modules (clusters), which ranged in size from 46 to 11,082 genes with a mean size of 1546 genes (Figure 5A and Table S7). In total, 3446 TFs (out of 5776) were assigned to modules and these made up on average 5.9% of genes within each module (Figure 5B). In total, 259 NACs (out of 453) were assigned to 23 of the 37 modules (Figure 5C). NAC TFs were overrepresented (χ^2^ *P* < 0.05) within modules 1, 6, 20, 29, and 34, respectively, as 11, 12, 17, 31, and 21% of all TFs in those modules were NACs compared to an average across all modules of 8%. We carried out GO term enrichment on all genes within these modules and found that these modules are enriched for phosphorylation (module 1), exocytosis and cell wall organization (module 6), protein export from the nucleus and response to water (module 20), photosynthesis (module 29), and regulation of photoperiodism and flowering (module 34) (Table S8). In general NACs within coexpressed modules were from several phylogenetic groups (Figure 5D). However, certain modules, *e.g.*, 17, 20, 26, and 29, contained genes from only one group (b, d, c, and a, respectively). These modules were enriched for GO terms related to response to heat and abiotic stress (module 17), protein export from nucleus and response to water (module 20), protein phosphorylation and system development (module 26), and photosynthesis (module 29). This indicates that some phylogenetically related NACs share similar expression profiles and may be involved in regulating similar biological processes. Interestingly, module 20 and 29 were both enriched in NACs compared to other TFs and specifically in NACs from groups d and a, respectively. This indicates that NACs may play a relatively major role in the regulation of these processes given their overrepresentation compared to other TFs in these coexpressed modules.

## Discussion

The availability of a more complete genome sequence for wheat has allowed the comprehensive analysis of wheat TF families. We identified 5776 TF genes, which is 1.5–3-fold higher than has previously been reported for wheat [3820 in wDBTF (Romeuf *et al.* 2010), 2407 in WheatTFDB (Chen *et al.* 2015), and 1940 in PlantTFDB (Jin *et al.* 2014)]. We found that, overall, wheat has 5.1 times more TFs than in other diploid Triticeae species. Compared to rice, wheat has 3.1 times more TFs as would be expected for a hexaploid species. The incomplete nature of the genomes of other monocots may explain the higher than expected ratio (3:1) of wheat TFs to monocots other than rice. Each family is present in wheat in similar proportions to those found in other monocots. In the future, it will be of value to compare the TF families found in the Chinese Spring reference sequence, used in this study, to TFs found in other wheat varieties. TFs are likely to vary in number and sequence between varieties; for example the MADS box TF *VRN-A1* varies in copy number, which influences flowering time (Díaz *et al.* 2012). Global comparisons will become possible as additional varieties are sequenced and a wheat pan genome is established [reviewed in Uauy (2017)]. A gold standard RefSeqv1.0 assembly will shortly become available for the wheat genome alongside new gene models. This new annotation may alter the exact numbers of TFs; however, the TGAC gene models are highly complete and we do not expect large changes. The TFs identified in this study, and most genome-wide studies, are *in silico* predictions based on gene sequence and domain content: therefore, further biological experiments will be required to confirm their sequence, gene structure, and function as TFs.

The NAC family is one of the largest TF families and has been characterized previously in other species (Ooka *et al.* 2003; Christiansen *et al.* 2011; Nuruzzaman *et al.* 2010; Peng *et al.* 2015; Saidi *et al.* 2017; Le *et al.* 2011). However, this is the first study to identify the NAC genes in hexaploid wheat and characterize their global expression patterns. We found that NAC TFs were located across all chromosomes, but were most frequently found on chromosome group 2 (on average 39 NACs per homeolog) with relatively few NACs on group 1 (on average seven NACs per homeolog). The uneven distribution of NACs across chromosomes has also been observed in rice (Nuruzzaman *et al.* 2010) and maize (Peng *et al.* 2015). In wheat, three homeologous copies of each gene (triads) would be expected due to its hexaploid genome. We found that, for most NACs, a complete triad (single copy of each homeolog) has been retained, although in one-third of triads one homeologous copy has been lost. This study of the NAC TFs is one of the first analyses in wheat of a whole gene family using a highly complete reference sequence, therefore further work will be required to find out whether the NACs are representative of homeolog conservation throughout the genome. However, unequal preservation of homeologs is supported by the analysis across all TF families in which the numbers of each TF family found per homeolog are frequently different (Table S3). This suggests that some gene loss or gain may have occurred in specific homeologs in many TF families. It is also possible that some gene loss may be explained by varietal differences or the incomplete nature of the reference sequence.

We found that wheat NAC TFs belong to eight main phylogenetic groups, similar to *Arabidopsis*, rice, and barley. Wheat has a reduced f group with only 13 genes compared to the 10 genes found in rice, but not compared to barley, suggesting that group f NACs were reduced in number in the ancestral Triticeae. This family-specific reduction requires further investigation to determine its biological relevance.

The DNA- and protein-binding NAC domain of NAC TFs has been studied over the past two decades (Xie *et al.* 2000; Welner *et al.* 2012; Ernst *et al.* 2004); however, the function of the CTD remains poorly understood. We detected previously identified CTD motifs in wheat, rice, and barley NAC TFs and also identified three novel CTD motifs. These motifs were in general restricted to one or two NAC groups. The presence of these motifs was typically conserved within closely related clades of rice, barley, and wheat orthologs. This is expected given the high overall sequence similarity between orthologs in these species. However, the conservation of CTD motifs extends beyond the immediate orthologs in these species. For instance, motifs iii and xiii in group c are conserved across several discrete clades that contain rice, barley, and wheat members. This evolutionary conservation inside otherwise nonconserved regions indicates that CTD motifs may have important biological functions. The *de novo* identification of CTD motifs that match those identified in studies of other plant species also highlights the conservation of motifs within angiosperms and indeed the plant kingdom as a whole (Pereira-Santana *et al.* 2015; Shen *et al.* 2009; Ooka *et al.* 2003). These motifs are, thus, good candidates for further investigation into the role of the NAC CTD and the specific function of these motifs.

In this study, we also combined global gene expression data from 308 RNA-seq samples with TF annotations. We found that, within the phylogenetic groups a–h, there are variations in expression patterns, although there are clades of genes that have extremely similar patterns. These genes with conserved expression patterns in particular tissues may represent good candidates to explore for functional roles in those tissues. In rice, for example, the use of coexpression as a guide to putative function has been successful in identifying several TFs regulating grain filling (Xu *et al.* 2016; Fu and Xue 2010), suggesting that this method might also prove useful in wheat. Sequenced mutant populations (Krasileva *et al.* 2017) and gene editing methods (Wang *et al.* 2014; Liang *et al.* 2017; Zhang *et al.* 2016) provide a direct route for hypothesis testing.

We produced coexpression modules that can be used to inform a range of further studies. Focusing on wheat NAC TFs, we found several examples where GO term enrichment of coexpressed genes supports known TF function. For example, *TaNAC-S* was found to be coexpressed with genes related to photosynthesis (module 2) according to GO term enrichment. It has previously been shown that *TaNAC-S* overexpression delays senescence and increases the expression of Rubisco, which is a central enzyme for carbon fixation in photosynthesis (Zhao *et al.* 2015). *TaNAM1* and *TaNAM2* were found in module 9, which is enriched for protein ubiquitination-related genes. *TaNAM* genes are known to increase protein content in the grain by increasing the remobilization of nitrogen from vegetative tissues (Waters *et al.* 2009). The ubiquitin pathway has previously been linked to senescence (Vierstra 2003), and several e3 ubiquitin ligases are downregulated in *TaNAM1* and *TaNAM2* mutants (Pearce *et al.* 2014) indicating that these genes may act through the ubiquitin pathway to bring about protein degradation for remobilization during senescence. Several NAC TFs including *TaNAC2* and *TaNAC4* have been reported to be responsive to both abiotic and biotic stresses (Xia *et al.* 2010a; Mao *et al.* 2012; He *et al.* 2015), and their coexpression with genes involved in protein phosphorylation (module 1) may provide a putative mechanism as to how they regulate responses to multiple stresses. These examples indicate that our coexpression modules categorize known genes with appropriate GO terms. GO term enrichment may also be predictive of the functions of novel genes (Eisen *et al.* 1998). For example, in *Arabidopsis thaliana*, a zinc finger TF (*AtZFP2*) was predicted to regulate abscission due to its expression within a group of genes that had GO terms associated with cell wall modifying proteins, extracellular regulators, and TFs. *AtAFP2* was subsequently demonstrated to regulate abscission in overexpression lines (Cai and Lashbrook 2008).

Previously characterized wheat NAC TFs were only identified in five coexpression modules out of the total 23 modules in which NAC TFs were expressed. This indicates that NAC TFs may still play unrecognized roles in wheat. This study provides the framework for further investigations of NAC TF function in this important crop species.

## Supplementary Material

Supplemental material is available online at www.g3journal.org/lookup/suppl/doi:10.1534/g3.117.043679/-/DC1.
