# Supplementary figures and images for: Genome-Wide Sequence and Expression Analysis of the NAC Transcription Factor Family in Polyploid Wheat
Source: G3 (Bethesda). 2017 Jul 11;7(9):3019–29. doi: 10.1534/g3.117.043679 (PMC5592928; doi:10.1534/g3.117.043679)

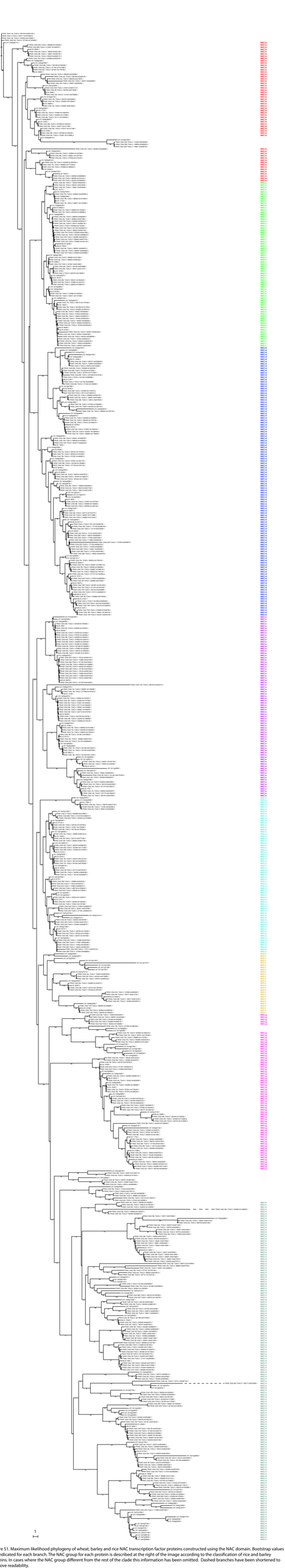

Supplement: Supplementary file 1 [file 3019FigureS1.pdf]
