# Supplementary material for: Genome-Wide Sequence and Expression Analysis of the NAC Transcription Factor Family in Polyploid Wheat
Source: G3 (Bethesda). 2017 Jul 11;7(9):3019–29. doi: 10.1534/g3.117.043679 (PMC5592928; doi:10.1534/g3.117.043679)
Supplement: Supplementary file 4 [file 3019TableS1.docx]

Table S1. NAC protein C-terminal domain motifs identified by Ooka et al. 2003

| **Group** | **Gene subgroups** | **Motif(s)** |
| --- | --- | --- |
| i | TERN | [W/N]XWEQX[N/T]W |
| ii | NAP | IFSDXXXXXXIYDGG; YQIPGLNWY |
| iii | AtNAC3, OsNAC7 | LP[Q/X]L[E/X]S[P/A] |
| iv | AtNAC3 | SXXXXDVLXSXXXEI; FDWAXXXG; NXXPELXXXXXXP; FG[Y/V]SGXQ |
| v | ATAF | EVQS[E/X]PK[W/I] |
| vi | OsNAC3 | WGETRTPESXXVD[N/A]D[P/A]FPE[L/M]D |
| vii | NAC2 | SXFXXALXXLXXSXPSXPAXAXEXXXLNXTXXRXS |
| viii | TIP | QGXX[P/A][R/Q]R[L/A]RLQ[S/X][N/E] |
| ix | NAC1, NAM | LPPLXD; [E/X][H/X]VXCFS[N/X] |
| x | NAC1, NAM | [E/X][H/X]VXCFS[N/X] |
| xi | NAM | LPPLXD |
| xii | ANAC001 | MXXX[R/Y][S/N][D/N][H/Y][R/T]PX[K/N]X[L/V][T/S]G[V/I]XXD |
| xiii | ONAC001 | PSS(XXXX)SSCVT |

Motifs and corresponding NAC transcription factor subgroups from Ooka et al. 2003, Figure 4. Motifs were converted to standard REGEX format, with the exception of motif xiii where the parenthesized “X”s represent a stretch of unknown amino acids ranging from 0 to 4 residues in length.
