# Supplementary material for: Genome-Wide Sequence and Expression Analysis of the NAC Transcription Factor Family in Polyploid Wheat
Source: G3 (Bethesda). 2017 Jul 11;7(9):3019–29. doi: 10.1534/g3.117.043679 (PMC5592928; doi:10.1534/g3.117.043679)
Supplement: Supplementary file 9 [file 3019TableS6.docx]

Table S6. *De novo* motif discovery in NAC groups.

| **Group** | **Motif** | **E-value*** | **Ooka et al. 2003** | **Pereira et al. 2015** | **Shen et al. 2009** |
| --- | --- | --- | --- | --- | --- |
| a | 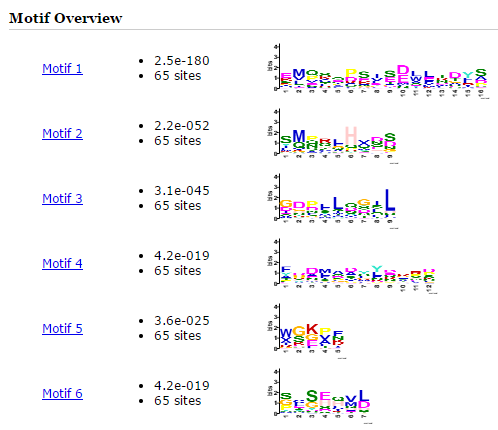 | 2.5e-180 | - | - | - |
| b | 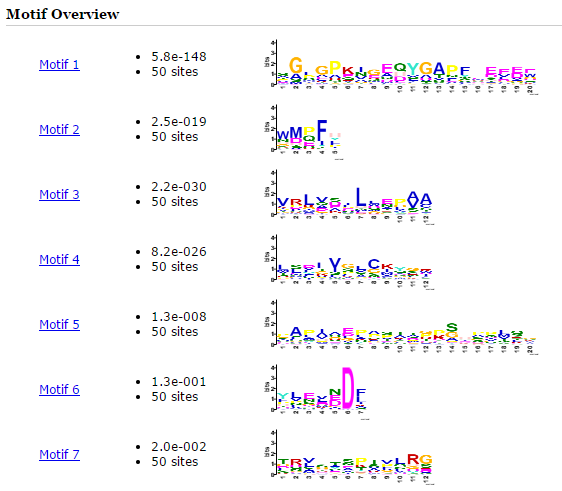 | 5.8e-138 | - | Group 3** | - |
| c | 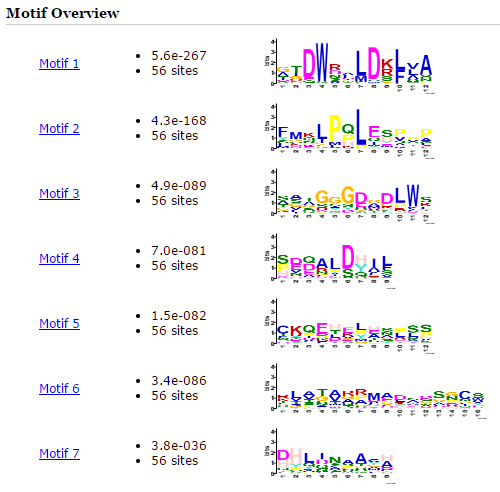 | 5.6e-267 | - | Group 1 | C.M6 |
| c | 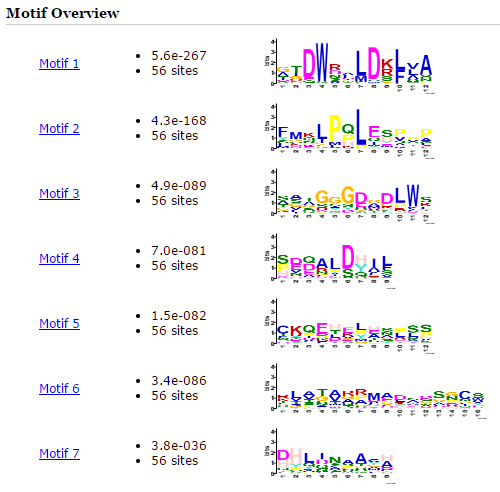 | 4.3e-168 | Group iii (AtNAC3; OsNAC7) | - | C.M7 |
| d | 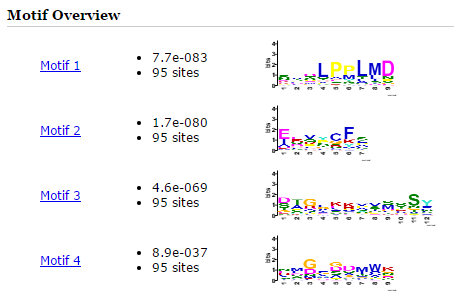 | 7.7e-083 | Group xi (NAM) | Group 2 | - |
| e | 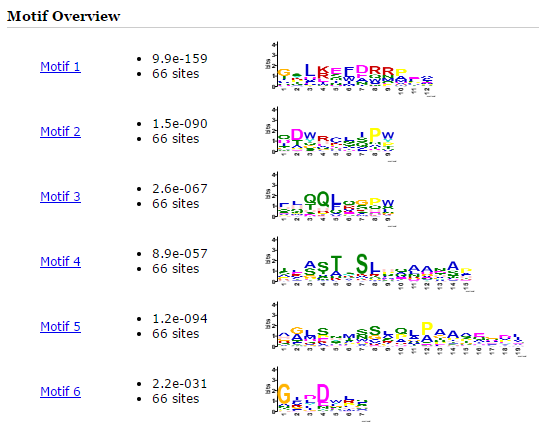 | 9.9e-159 | - | - | - |
| f | 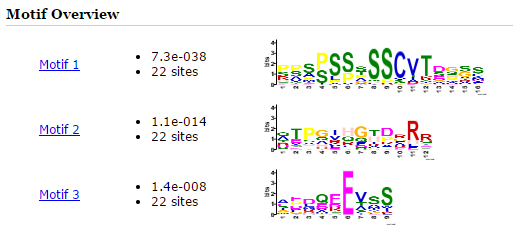 | 7.3e-038 | Group xiii (ONAC001) | - | - |
| g | 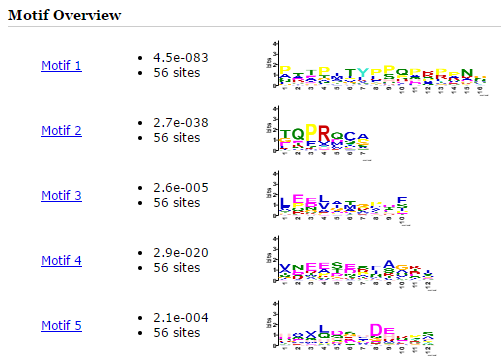 | 4.5e-083 | - | - | - |
| h | 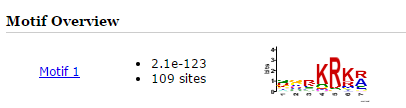 | 2.1e-123 | - | Group 5**^ | - |

*Statistical significance of the motif, based on the estimated number of motifs with the same width, site count and log likelihood ratio expected to be found in a similarly sized set of sequences.

**Found *de novo* from Pereira et al. 2015 raw data; not listed in paper.

^Not significant in the Pereira et al. 2015 Group 5 output (no motifs were significant), but similar motif arrangement.
