# Supplementary material for: Genome-Wide Sequence and Expression Analysis of the NAC Transcription Factor Family in Polyploid Wheat
Source: G3 (Bethesda). 2017 Jul 11;7(9):3019–29. doi: 10.1534/g3.117.043679 (PMC5592928; doi:10.1534/g3.117.043679)
Supplement: Supplementary file 11 [file 3019TableS8.docx]

Table S8. Most over represented biological process GO terms in co-expression modules. Revigo was used to summarise GO terms using the settings medium similarity, p-values, GO term database Oryza sativa, semantic similarity SimRel. N/A indicates no over represented biological process GO terms were identified.

| **Module** | **Most over represented biological processes** | **p-value (GO term 1)** | **p-value (GO term 2)** |
| --- | --- | --- | --- |
| 1 | phosphorylation, protein phosphorylation | 1.41E-96 | 4.49E-93 |
| 2 | photosynthesis, carotenoid biosynthesis | 9.32E-110 | 1.05E-30 |
| 3 | protein ubiquitination, transcription regulation | 1.87E-18 | 1.01E-14 |
| 4 | microtubule organisation, DNA replication | 1.46E-49 | 5.52E-48 |
| 5 | xylem development, cinnamic acid biosynthetic process | 1.02E-09 | 8.72E-09 |
| 6 | exocytosis, cell wall organisation | 3.90E-03 | 1.78E-02 |
| 7 | DNA integration, telomere maintenance | 1.06E-48 | 3.67E-21 |
| 8 | carbohydrate biosynthesis, signalling | 5.84E-20 | 1.55E-16 |
| 9 | transcription regulation, protein ubiquitination | 2.60E-12 | 9.49E-11 |
| 10 | intracellular protein transport,  microtubule-based movement | 8.67E-06 | 8.67E-06 |
| 11 | translation initiation, glycolytic process | 8.98E-06 | 5.67E-03 |
| 12 | translation, ribosome biogenesis | 5.04E-17 | 8.01E-16 |
| 13 | rRNA processing, translation | 2.70E-39 | 2.91E-38 |
| 14 | translation, translational initiation | 1.00E-300 | 6.07E-18 |
| 15 | cell wall organisation, metabolism | 1.44E-05 | 5.03E-05 |
| 16 | ubiquitin dependent protein catabolism, protein ubiquitination | 3.11E-75 | 1.61E-54 |
| 17 | response to heat, response to abiotic stress | 3.43E-14 | hydrogen peroxide (1.55E-08),  heat (3.88E-06), high light (5.50E-06) |
| 18 | sucrose metabolism, iron-sulfur cluster assembly | 1.23E-03 | 1.56E-02 |
| 19 | telomere maintenance, protein import | 1.05E-02 | 1.05E-02 |
| 20 | protein export from nucleus, response to water | 6.15E-05 | 1.17E-04 |
| 21 | ion transmembrane transport,  metabolic process | 2.67E-05 | 1.13E-03 |
| 22 | cellulose biosynthesis, cell wall organisation | 1.06E-04 | 1.06E-04 |
| 23 | positive regulation of programmed cell death | 8.45E-04 | N/A |
| 24 | phosphatidylethanolamine binding, protein dimerization activity | 1.85E-03 | 1.94E-03 |
| 25 | pectin catabolism, cell wall modification | 1.16E-06 | 4.16E-04 |
| 26 | protein phosphorylation, system development | 6.26E-05 | 1.46E-04 |
| 27 | hydrogen ion transmembrane transport, protein catabolism | 9.72E-09 | 6.02E-07 |
| 28 | mRNA splicing, seed dormancy process | 2.55E-05 | 2.55E-05 |
| 29 | photosynthesis light reaction, photosynthetic electron transport | 2.31E-23 | 3.90E-21 |
| 30 | decapping of nuclear-transcribed mRNA, transcription | 1.39E-02 | 1.69E-02 |
| 31 | translation, translation elongation | 5.79E-72 | 6.79E-12 |
| 32 | vitamin B6 biosynthesis, pyridoxal phosphate biosynthetic process | 4.35E-03 | 9.07E-03 |
| 33 | N/A | N/A | N/A |
| 34 | regulation of photoperiodism flowering, multicellular organism development | 6.37E-03 | 1.58E-02 |
| 35 | ATP synthesis coupled electron transport, aerobic respiration | 7.04E-08 | 1.82E-06 |
| 36 | phosphorelay signal transduction | 1.36E-04 | N/A |
| 37 | N/A | N/A | N/A |
