# Supplementary material for: Genome-Wide Sequence and Expression Analysis of the NAC Transcription Factor Family in Polyploid Wheat
Source: G3 (Bethesda). 2017 Jul 11;7(9):3019–29. doi: 10.1534/g3.117.043679 (PMC5592928; doi:10.1534/g3.117.043679)
Supplement: Supplementary file 2 [file 3019FigureS2.pdf]

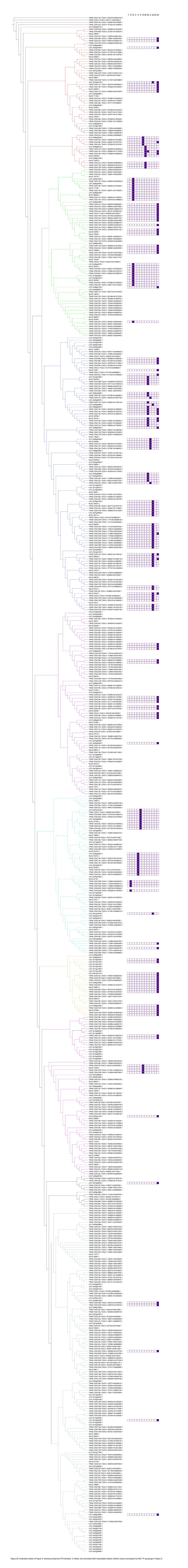

Figure S2. Extended version of Figure 3, showing conserved CTD domains in wheat, rice and barley NAC transcription factors. Branch colours correspond to NAC TF groupings in Figure 2.
