# Supplementary material for: Genome-Wide Sequence and Expression Analysis of the NAC Transcription Factor Family in Polyploid Wheat
Source: G3 (Bethesda). 2017 Jul 11;7(9):3019–29. doi: 10.1534/g3.117.043679 (PMC5592928; doi:10.1534/g3.117.043679)
Supplement: Supplementary file 3 [file 3019FigureS3.pdf]

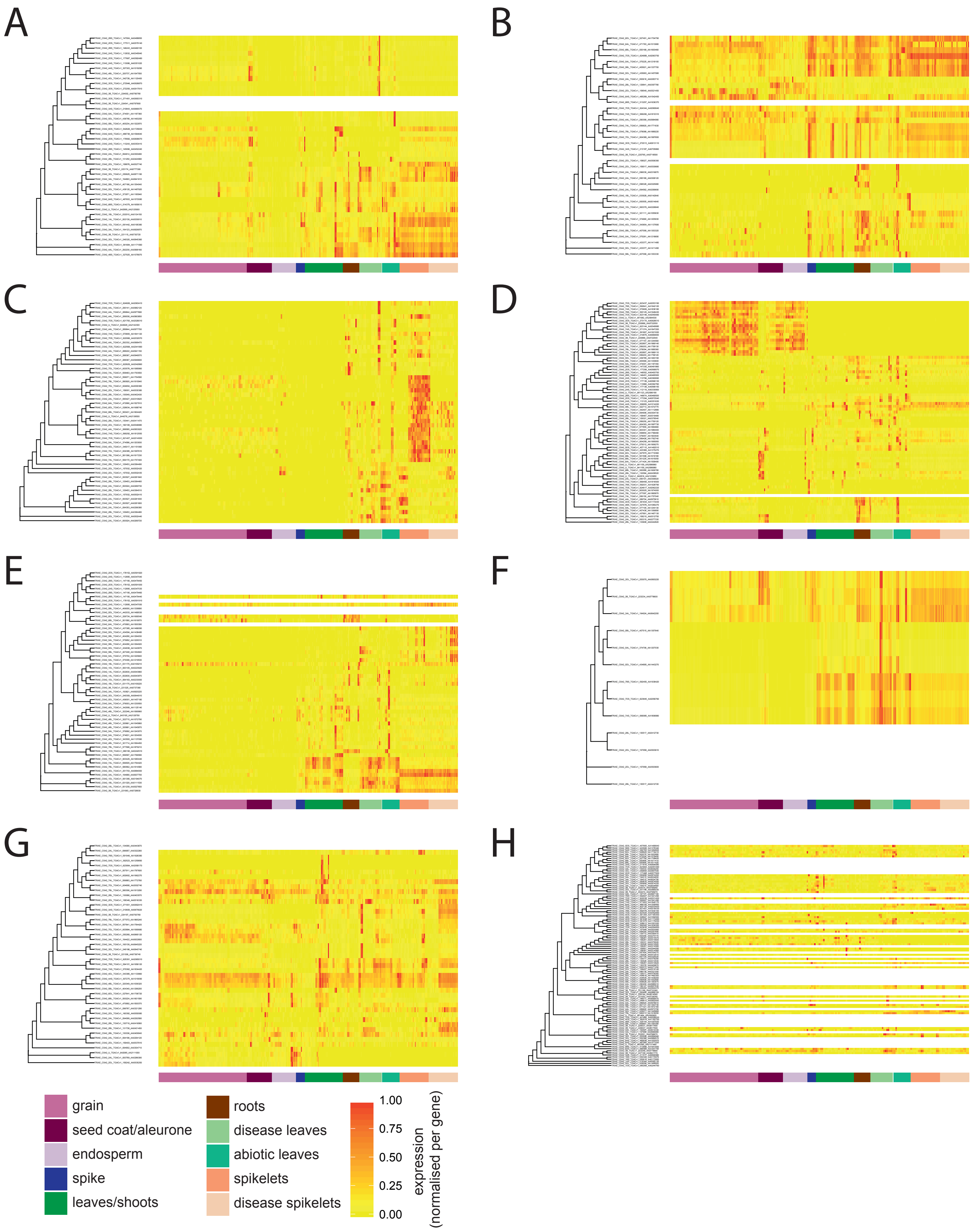

Figure S3. Extended version of Figure 4 with gene IDs. Relationship between phylogenetic position and NAC gene expression across 308 RNA-seq samples from diverse tissues, developmental stages and stress conditions. The origin of each sample is indicated by the coloured bar under each heatmap. Each panel (A-H) represents NAC genes belonging to that group according to the classification in Figure 2. Dendrograms indicate the maximum likelihood phylogeny of genes within each group. Genes which did not meet the minimum expression criteria (>0.5 tpm in at least three samples) do not have expression data represented (white rows). All other expression data (tpm) was normalised per gene to range from 0 to 1.
